# Disruption of transfer entropy and inter-hemispheric brain functional connectivity in patients with disorder of consciousness

**DOI:** 10.3389/fninf.2013.00024

**Published:** 2013-11-13

**Authors:** Verónica Mäki-Marttunen, Ibai Diez, Jesus M. Cortes, Dante R. Chialvo, Mirta Villarreal

**Affiliations:** ^1^Department of Cognitive Neuroscience, Institute for Neurological Research, FLENIBuenos Aires, Argentina; ^2^CONICETBuenos Aires, Argentina; ^3^Biocruces Health Research Institute, Hospital Universitario de CrucesBarakaldo, Spain; ^4^Ikerbasque, The Basque Foundation for ScienceBilbao, Spain

**Keywords:** disorder of consciousness, resting state, functional magnetic resonance imaging, BOLD signal, transfer entropy, partial correlation, functional connectivity, brain networks

## Abstract

Severe traumatic brain injury can lead to disorders of consciousness (DOC) characterized by deficit in conscious awareness and cognitive impairment including coma, vegetative state, minimally consciousness, and lock-in syndrome. Of crucial importance is to find objective markers that can account for the large-scale disturbances of brain function to help the diagnosis and prognosis of DOC patients and eventually the prediction of the coma outcome. Following recent studies suggesting that the functional organization of brain networks can be altered in comatose patients, this work analyzes brain functional connectivity (FC) networks obtained from resting-state functional magnetic resonance imaging (rs-fMRI). Two approaches are used to estimate the FC: the Partial Correlation (PC) and the Transfer Entropy (TE). Both the PC and the TE show significant statistical differences between the group of patients and control subjects; in brief, the inter-hemispheric PC and the intra-hemispheric TE account for such differences. Overall, these results suggest two possible rs-fMRI markers useful to design new strategies for the management and neuropsychological rehabilitation of DOC patients.

## 1. Introduction

Recent studies have shown that brain networks obtained from functional Magnetic Resonance Imaging (fMRI) recordings are altered in patients with severe disorder of consciousness (DOC) (Boveroux et al., 2010; Noirhomme et al., 2010; Heine et al., 2012; Perri et al., 2013). DOC can result from severe brain injury and is characterized by an absence of awareness of the self and the environment, either with preserved or disrupted sleep-awake cycle. DOC encompasses a wide spectrum of clinical conditions with different levels in the content of conscious awareness, ranging from the coma state (CS, patients who have a disrupted sleep-awake cycle and don't wake up), vegetative state (VS, who preserve sleep-awake cycle but are unaware of themselves and the environment), minimally consciousness state (MCS, patients who are unable to reliably communicate but show reproducible albeit fluctuating behavioral evidence of awareness), to lock-in syndrome (LI, patients who are fully conscious but are completely paralyzed except for small movements of the eyes or eyelids). For the prognosis of these patients, the clinical practice scores this graduation in DOC response by the Glasgow Coma Scale (GCS) (Teasdale and Jennett, 1974), or as we will use in this paper, by an alternative scale such as the JFK Coma Recovery Scale-Revised (CSR-R) (Giacino et al., 2004). This scale encodes the neurological and behavioral state of the DOC patient providing a number ranging from 0 to 23, 0 for the deepest coma state, 23 for the fully recovered one. Despite the existence of such scales, there is a need for more reliable methods that based on brain neuroimaging can provide better characterization of the large-scale disturbances of brain function in DOC. Ultimately these approaches should help in understanding and eventually predicting coma outcome.

The resting state functional Magnetic Resonance Imaging (rs-fMRI) accounts for the spontaneous brain activity occurring in the high-amplitude ultra-slow (0.1 Hz) fluctuations in the Blood-Oxygen-Level-Dependent (BOLD) signal, defining networks of correlated spontaneous activity of brain Functional Connectivity (FC) (Raichle et al., 2001; Beckmann et al., 2005). The interaction between these distributed networks as well as subcortical modules is considered critical for conscious processing, and has been shown to be disrupted in DOC state (Tononi, 2004; Cauda et al., 2009; Rosanova et al., 2012). Furthermore, the rs-fMRI paradigm is a very suitable strategy for DOC patients, since they are not able to efficiently perform specific tasks. The present study addresses the question of whether the FC obtained from the rs-fMRI is altered at different brain regions as a consequence of consciousness disturbances. To this end, we investigate the FC obtained by two different measures: the Partial Correlation (PC) and the Transfer Entropy (TE), in two different groups: healthy adults and DOC patients.

Information theory offers an arsenal of different measures, complementing the linear correlation estimations of FC. These information tools are typically built as extensions of the Shannon Entropy, quantify the interactions between variables by measuring the information which is shared or transferred between them (Jaynes, 1957; Cover and Thomas, 2006). In the last decade, the TE method is growing in popularity as it can account for directed interactions between time-series variables (Schreiber, 2000). When applied to neuroimaging time-series, TE is a data-driven measure that assesses the functional connectivity between brain areas even for non-linear interactions. Unlike the correlations, TE reveals directionality in the interactions, allowing for determining a *directed* FC between areas.

We hypothesize that FC would be reduced in DOC patients since consciousness implies functional integration (Tononi, 2004). We anticipate that PC and TE would show different behaviors in patients with increasing level of consciousness, provided that they can be related to different mechanisms of information processing in the brain.

The paper is organized as follow: in Material and Methods, we give details on the the data acquisition and preprocessing and define the two measures PC and TE to compute FC patterns. The next section is dedicated to present the results of the analysis. The paper closes with a discussion on some consequences of the alteration of the FC patterns in DOC patients.

## 2. Materials and methods

### 2.1. Subjects

Seventeen healthy subjects (**Group 1**) aged 25 ± 5 year old (8 men, 9 women), with no history of neurological or psychiatric problems, participated in this study as a control group. The Edinburgh Handedness Inventory was used to assess handedness (Oldfield, 1971), resulting in thirteen subjects right-handed and four left-handed. Eleven DOC patients (**Group 2**) were scanned (age range, 17–44 years; 6 men, 5 women). Data from two patients were subsequently excluded because of unacceptable degrees of head and body movements. The coma severity for each patient was clinically assessed using the Revised Coma Recovery Scale [CRS-R, (Giacino et al., 2004)]: scores range from 0 (meaning deep coma state) to 23 (full recovery). The patients were scanned the first time between 2 to 6 months after major acute brain injury, and a second time between 3 to 6 months after the first scan (Table 1). For better comparison, group 2 was subdivided into 2 subgroups: **Group 2a** (*n* = 12) is composed by all scans of DOC patients who had a corresponding CRS-R scale. **Group 2b** (*n* = 4) includes the second scans of the four patients who recovered consciousness before the second session (marked with asterisks in Table 1). The study protocol was approved by the Institutional Review Board of the Institute of Neurological Research FLENI. Informed consent was directly obtained from healthy participants and from the next kin of each of the patients.

**Table 1:** **Clinical characteristics of DOC patients**.

**Patient code**	**Age**	**Time between accident and first scan (months)**	**Clinical assessment at first scan**	**Time between first and second scan (months)**	**Clinical assessment at second scan**
P1	34	2	VS	5	VS
P2^*^	18	4	MCS	4	C
P3^*^	44	2	MCS	3	C
P4	17	6	VS	6	MCS
P5	26	4	VS	3	MCS
P6^*^	26	4	EMCS	4	C
P7	29	4	MCS	3	MCS
P8	41	2	VS	6	VS
P9^*^	34	5	VS	5	C

* *Patients that recovered from DOC at the second scan. VS, Vegetative State; MCS, Minimally Consciousness State; C, Conscious; EMCS, Emergence from MCS (an intermediate state between MCS and C)*.

### 2.2. MRI data acquisition and preprocessing

The fMRI measurements were carried out on a 3T Signa HDxt GE scanner using an 8 channel head coil. Change in blood-oxygenation-level-dependent (BOLD) T2^*^ signal was measured using an interleaved gradient-echo EPI sequence. Thirty contiguous slices were obtained in the AC-PC plane with the following parameters: 2 s repetition time (TR), flip angle: 90°, 24 cm field of view, 64 × 64 pixel matrix, and 3.75 × 3.75 × 4.0 mm voxel dimensions. During the experimental session subjects lied quietly for a period of 7 min. 220 whole brain volumes were obtained per scan session, including 5 dummy scans to allow for T1 saturation effects that were discarded from the analysis. High resolution T1-weighted 3D fast SPGR-IR were also acquired (*TR* = 6.604 ms, *TE* = 2.796 ms, *TI* = 450; parallel imaging (ASSET) acceleration factor = 2; acquisition matrix size = 256 × 256; FOV = 24 cm; slice thickness = 1.2 mm; 120 contiguous sections). The image data was analyzed using SPM8 (Wellcome Department of Cognitive Neurology, London, UK) implemented in MATLAB (MathWorks Inc., Natick, MA). The functional images were subjected to temporal alignment and volumes were corrected for movement using a six-parameter automated algorithm. The realigned volumes were spatially normalized to fit to the template created using the Montreal Neurological Institute reference brain based on Talairach and Tournoux's sterotaxic coordinate system (Ashburner and Friston, 1999). The spatially normalized volumes consisting of 4 × 4 × 4 mm^3^ voxels were smoothed with a 8-mm FWHM isotropic Gaussian kernel. Additionally, a linear trend removal and band pass filtering between 0.01 and 0.08 Hz was applied on the data.

### 2.3. Brain parcellation and regions of interest

**Figure 1:**
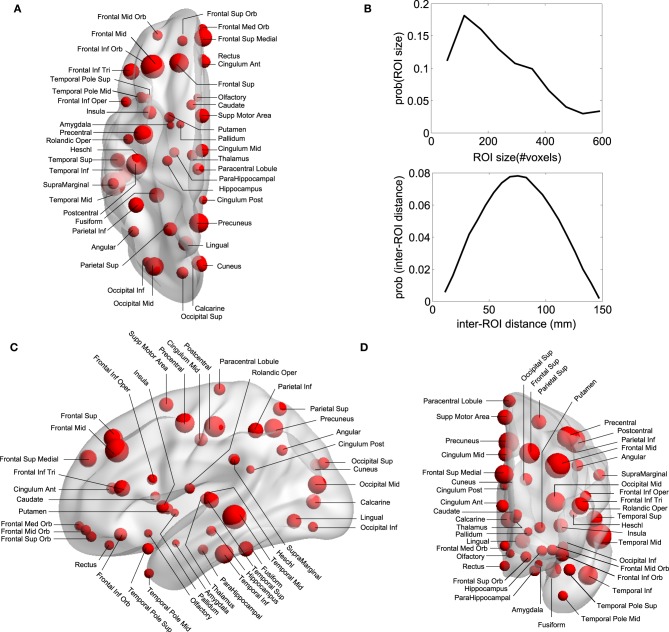
**Anatomical Brain parcellation and Regions of Interest (ROI). (A)** Axial, **(C)** Saggital, **(D)** Coronal views. Specific ROI are depicted with spheres with diameters proportional to the ROI size (i.e., the number of voxels). Notice that the atlas has both cortical and subcortical components. **(B)** ROI size' distribution and inter-ROI distance' distribution. To give an estimation, as each voxel is about 4 cubic millimeters (see Materials and Methods), the ROI average size (≈150 voxels) is equivalent to a 3D cube of 21 mm edge. Biggest ROI (≈600 voxels) corresponds to 3D cubes of 34 mm edge.

Regions of Interest (ROI) were defined following the Automatic Anatomical Labeling (AAL) atlas (Tzourio-Mazoyer et al., 2002) (see Figures 1A–D) which comprises 90 different areas, 45 on each hemisphere (e.g., hippocampus Left, hippocampus Right, amygdala Left, amygdala Right, etc.). Importantly for the study of DOC patients, the AAL atlas includes both cortical and subcortical components (eg., hippocampus, thalamus and amygdala). Per each ROI we have extracted a mesoscopic (multi-voxel) fMRI time-series resulting from averaging over all fMRI time-series of all voxels within a given ROI (Figure 1B is showing the ROI size distribution among all areas). The MNI coordinates of the centroids in each ROI are used to calculate the Euclidean distance between each pair of regions (Figure 1B).

### 2.4. Functional connectivity matrices

Correlated areas in the rs-fMRI time series define the Functional Connectivity (FC) matrices. Two methods have been used to the FC: The PC and TE.

#### 2.4.1. The partial correlation

Matrix has dimensions 90 × 90 (with 90 the ROIs number) and each element is given by the pairwise PC between any two ROIs. PC is a correlation matrix that removes for a given ROIs pair the effect of the rest of the variables, i.e., removing the correlations contribution which are coming from common neighbors interactions. Let *C* be a non-singular correlation matrix, then each element of the PC matrix is given by

(1) $${\text{PC}}_{ij}=-\frac{P_{ij}}{\sqrt{P_{ii}P_{jj}}}$$

where *P*≡ *C*^−1^ is the inverse of the correlation matrix (ie., the precision matrix).

Notice that PC is a symmetrical measure, i.e., P*C*_*ij*_ = P*C_ji_*. We also have computed the standard correlations *C*, and although C is more noisy than PC, the results we are showing here for the PC are also valid for the standard correlation.

The PC was computed by using the *partialcorr* method incorporated in MATLAB (MathWorks Inc., Natick, MA). The second argument that the function *partialcorr* outputs is a matrix of p-values for testing the hypothesis of no PC against the alternative that there is a non zero PC.

PC matrices were calculated for each subject and grouped into the following categories: inter-hemispheric (between one area on the left and all the other areas at right hemisphere, or vice versa), homologous inter-hemispheric (one area on the left hemisphere and its homologous area on the right hemisphere, or vice versa), left intra-hemispheric, right intra-hemispheric, and total.

#### 2.4.2. Transfer entropy

quantifies the *directed* interaction between any two ROIs. To compute it, let define *i^F^* as the future of the time series in ROI *i*. Similarly, *i^P^* and *j^P^* the pasts of ROIs *i* and *j*. Then, the TE from *j* to *i* is defined as

(3) $${\text{TE}}_{ji}=H\left(i^{F}|i^{P}\right)-H\left(i^{F}|i^{P},j^{P}\right)$$

with *H*(*i^F^*|*i^P^*) = *H*(*i^F^*, *i^P^*) − *H*(*i^P^*), the conditional Shannon entropy of *i^F^* conditioning on *i^P^* [for details, see (Cover and Thomas, 2006)]. Similarly, *H*(*i^F^*|*i^P^*, *j^P^*) = *H*(*i^F^*, *i^P^*, *j^P^*) − *H*(*i^P^*, *j^P^*) is the conditional Shannon entropy of *i^F^* conditioning on *i^P^* and *j^P^*.

The TE is a non-symmetrical measure, i.e., TE_*ij*_ ≠ TE_*ji*_.

The Shannon Entropy (average uncertainty) of the random variable *X* is defined as *H*(*X*) = −∑_*x*_ prob(*x*)log prob(*x*), where *x* represents a possible state in variable *X* (Cover and Thomas, 2006). For base 2 logarithm (as we have done here), the information is expressed as information bits.

To compute probabilities from continuous variables, we did not perform binning; alternatively, we just rounded each value in the time series to its nearest integer and computed probabilities (number of time points in a given state divided by the total time-series length). The conditional entropies have been calculated with the function *condentropy* developed by Hanchuan Peng in C++ and plug-into MATLAB via mex. The code is available for download from Peng (Peng).

For the past of the time series it was considered the original time series. Their future were built by shifting the time series in MATLAB with the function *circshift* with a lag value of 10 time points. This lag number was previously chosen (and fixed for all simulations) in order to maximize TE values.

The statistical significance of the TE values was estimated by shuffling the time series of the target ROI (for the calculation of the TE from *j* to *i*, hereafter *j* will be referred as the source and *i* as the target). The time series was shuffled to remove the temporal information in the target variable. Next, the TE value is calculated for many repetitions of this shuffling procedure to obtain the distribution of values under the null hypothesis of zero values of TE (i.e., zero uncertainty reduction from source *j* to target *i*).

TE matrices were calculated for each subject and grouped into the following categories: homologous inter-hemispheric (one area on the left hemisphere to its homologous area on the right hemisphere and vice versa), left intra-hemispheric, right intra-hemispheric, inter-hemispheric (from one area on the left to all the other areas at right hemisphere, and from one area on the right to all other areas at the left hemisphere) and total.

#### 2.4.3. Summary of brain categories

For easy reading we have adopted the following notation:

- PC calculations: LR: inter- hemispheric (between one area on one hemisphere and all the other areas at the other hemisphere). As the PC calculation is symmetric (LR is the same than RL) we condensed the inter-hemispheric PC in only LR. HIH: homologous inter-hemispheric. LL: left intra-hemispheric. RR: right intra-hemispheric.
- TE calculations:
  HLR: homologous inter-hemispheric from left to right (one area on the left hemisphere to its homologous area on the right hemisphere).
  HRL: homologous inter-hemispheric from right to left (one area on the right hemisphere to its homologous area on the left hemisphere).
  LL: intra-hemispheric from left to left.
  RR: intra-hemispheric from right to right.
  LR: inter-hemispheric left-right (from one area on the left to all the other areas at right hemisphere).
  RL: inter-hemispheric right-left (from one area on the right to all the other areas at left hemisphere).

### 2.5. Statistical analysis

PC and TE individual matrices were thresholded at a probability value of 0.1 (i.e., 10% confidence); these data were used for Tables 2, 3 and all the figures shown in the paper. We also computed PC and TE matrices at different confidence values, 5% and 100% (zero threshold), and the results did not considerably change (cf. Tables S1–S4).

**Table 2:**
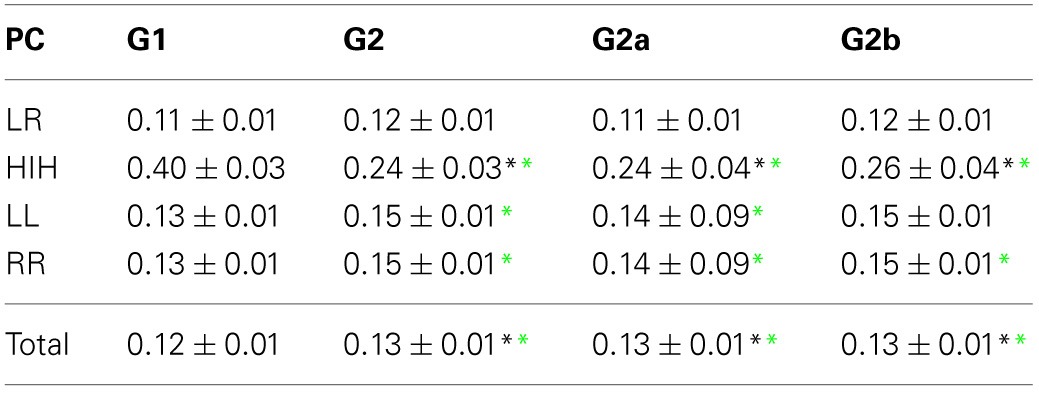
**PC average values ± standard deviation thresholded at 10% confidence (see Materials and Methods)**.

^*^Significantly different from G1; p < 0.05. Significant differences are indicated with black asterisks for ANOVA and green for Kruskal-Wallis tests. LR, inter-hemispheric; HIH, homologous inter-hemispheric; LL, left intra-hemispheric; RR, right intra-hemispheric.

**Table 3:**
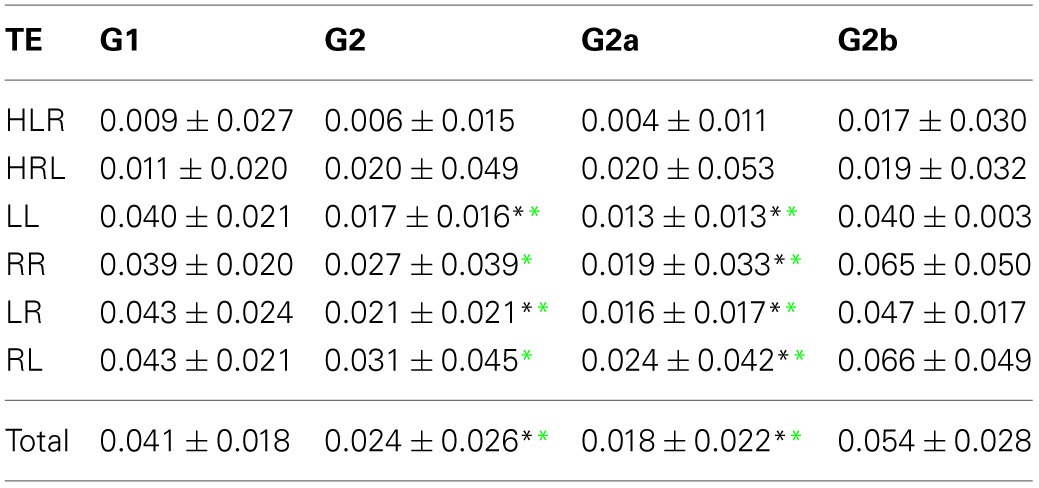
**TE average values ± standard deviation**.

^*^Significantly different from G1; p < 0.05. Significant differences are indicated with black asterisks for ANOVA and green for Kruskal-Wallis tests. HLR: homologous inter-hemispheric from left to right; HRL, homologous inter-hemispheric from right to left;

*LL, left intra-hemispheric; RR, right intra-hemispheric; LR, inter-hemispheric left to right; RL, inter-hemispheric right to left*.

For comparison of PC and TE values between the different brain categories and groups, a two-ways ANOVA test was performed, using the function *anovan* from MATLAB (MathWorks Inc., Natick, MA). For *post-hoc* analysis, multi-sample *t*-tests were performed between groups for each brain category using the function *multcompare* from MATLAB which include the Bonferroni correction for multiple comparisons. To assess possible deviations from the Gaussian distribution in the data, the Kruskal–Wallis non-parametric tests were also performed using the function *kruskal–wallis* from MATLAB. The groups comparison results showed very little differences across these tests, cf. Tables 2, 3, in which the statistically significant differences from control were denoted using asterisks at different colors (black for ANOVA and green for Kruskal–Wallis).

### 2.6. A further test for fMRI head motion artifacts

To reject the possibility of head motion artifacts, PC was re-computed in a matrix which included the original 90 ROIs from the AAL atlas plus two motion regressors: the translational modulus and rotational modulus. It is expected, if important correlations were introduced by head motion, that the PC results obtained from this expanded matrix must show significant differences in comparison with the results gathered from the original 90 ROI's. However, this was not the case; no changes were observed which indicates that the data is free of heat motion artifacts.

## 3. Results

### 3.1. Partial linear correlations (PC)

First we looked into the PC patterns (Table 2). ANOVA between G1 and G2 shows a significant effect of categories (*p* < 0.001), and a significant interaction between categories and groups (*p* < 0.001). Controls have a significantly smaller PC mean value than patients (*p* < 0.001). When looking into categories, HIH PCs are significantly higher than LL, RR, and LR (*p* < 0.001). In addition, LL and RR values are significantly higher than LR (*p* < 0.001). To further inspect the interaction, we performed *post-hoc* multiple comparison tests between groups for the different categories. HIH PCs are significantly higher in G1 (*p* < 0.001). The Kruskal–Wallis test gave the same results, with the addition of being LL and RR PCs significantly higher in G2 compared with G1 (*p* < 0.005).

**Figure 2:**
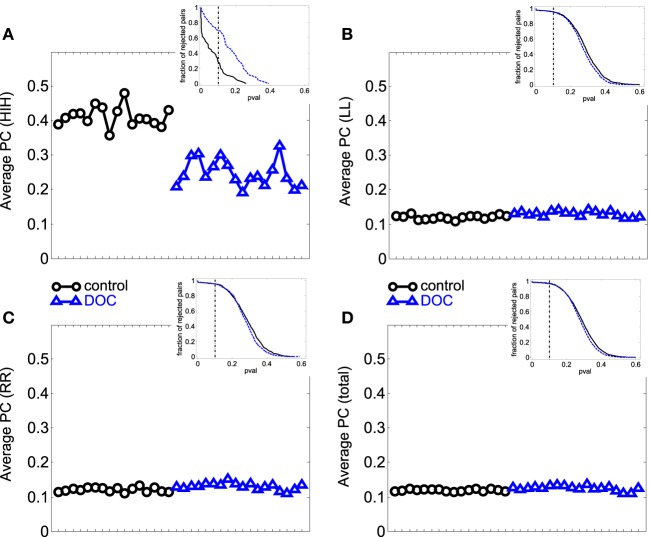
**Average PC values per subject. (A)** HIH (homologue inter-hemispheric areas); **(B)** LL (left intra-hemispheric); **(C)** RR (right intra-hemispheric); **(D)** total. Insets depict the fraction of rejected pairs of areas for a given probability level. PC values were thresholded at a probability value of 0.1 (dashed lines in the insets). Black circle: G1 (control); blue triangles : G2 (DOC). Observe the huge differences between G1 and G2 for HIH compared to LL and RR. For detailed values, see Table 2.

The comparison between G1 and G2a gives the same results. However, when comparing G1 and G2b, the effect of group still holds but is smaller than that between G1 and G2a (*p* = 0.002). The effect of categories is the same as in G1 vs. G2 comparison, and there is a significant interaction effect (*p* < 0.001). *Post-hoc* tests show that HIH PCs are significantly smaller in G2b with respect to G1 (*p* < 0.001). Finally, the comparison including all brain categories (total) was significant between G1 vs. G2 and G1 vs. G2a (*P* = 0.018 and 0.044 respectively). The same significant differences were conserved with the Kruskal–Wallis test. Results can be seen in Table 2 and Figure 2.

In summary, the partial linear correlations approach allows to expose a differential functional connectivity in a healthy conscious brain in comparison with a DOC state and a recent recovery from it. A reduced inter-hemispheric connectivity is evident in DOC patients.

### 3.2. Transfer entropy

We then examined the uncertainty reduction (information) transferred between ROIs pairs by computing the TE. ANOVA on TE values for G1 and G2 shows a significant effect of group (*p* = 0.0025) and categories (*p* < 0.001). Particularly there were significant differences between HLR and HRL TEs and the TE values for the other categories. In the case of HLR, TEs are significantly lower than LL, RR and inter-hemispheric (LR and RL) TEs (*p* < 0.005), whereas HRL TEs are significantly lower than RR and RL TEs (*p* < 0.025). There is no interaction effect between group and brain category. The *post-hoc* analysis showed that LL, LR and the total TE values differ between controls and patients.

However, when performing the ANOVA for G1 and G2a there is a significant effect of group (*p* < 0.001) and categories (*p* < 0.001). *Post-hoc* tests show that LL and RR TEs are significantly higher in G1 than in G2a (*p* < 0.05). In addition, TE for LR is also significantly higher in G1 (*p* = 0.001).

When comparing G1 and G2b, there was significant effect of group (*p* = 0.042) and categories (*p* < 0.001). Additionally, when looking into the main effect of brain sections, HLR and HRL TE values were significantly smaller than LR and RL (*p* < 0.05). However the multiple-compare test did not revealed any significant difference. The Kruskal–Wallis test gave the same general results but in this case adding significant differences between G2 and G1 in the same regions were we previously found only for G2a.

**Figure 3:**
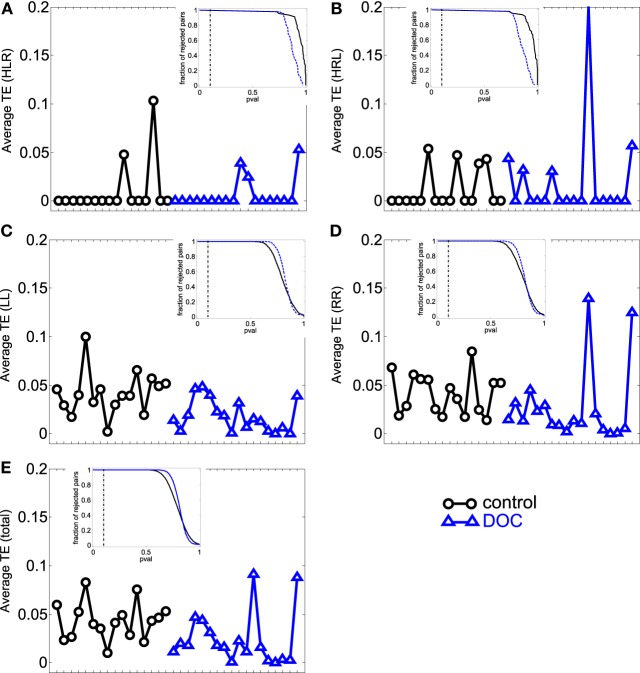
**Average TE values per subject. (A)** HLR (homologue left-right inter-hemispheric areas); **(B)** HRL (homologue right-left inter-hemispheric areas); **(C)** LL (left intra-hemispheric); **(D)** RR (right intra-hemispheric); **(E)** total. Insets depict the fraction of rejected pairs of areas for a given probability level. TE values were thresholded at a probability value of 0.1 (dashed lines in the insets). Black circles: G1 (control); blue triangles : G2 (DOC).

The results can be seen in Table 3 and Figure 3. If two time series are highly correlated, their TE is close to zero in both directions; if they are not correlated but one influences the other's behavior, TE is high in that direction and very low in the opposite direction. In our results, the significant smaller TE between homologue areas with respect to the other TE values is consistent to the fact that they are highly correlated (cf. results in 3.1).

The differences found within hemispheres between the groups parallelize the increased intra-hemispheric correlations in G2 and G2a. When looking at G2b group, their averages are also biased by one patient that presented extremely high TE values (corresponding to the last case in the x-axis).

In summary, TE analysis exposes alterations in the FC exhibited by DOC patients. In particular, TE within hemispheres and between hemispheres is smaller, although no difference was found when looking at homologue areas. In contrast to the results obtained in the PC analysis, the differences found uphold irrespective of the Euclidean distance separating ROIs pairs, although when considering LL TE, a slight decrease in the statistical p value can be observed.

### 3.3. Between-homologue inter-hemispheric PC and left intra-hemispheric TE

**Figure 4:**
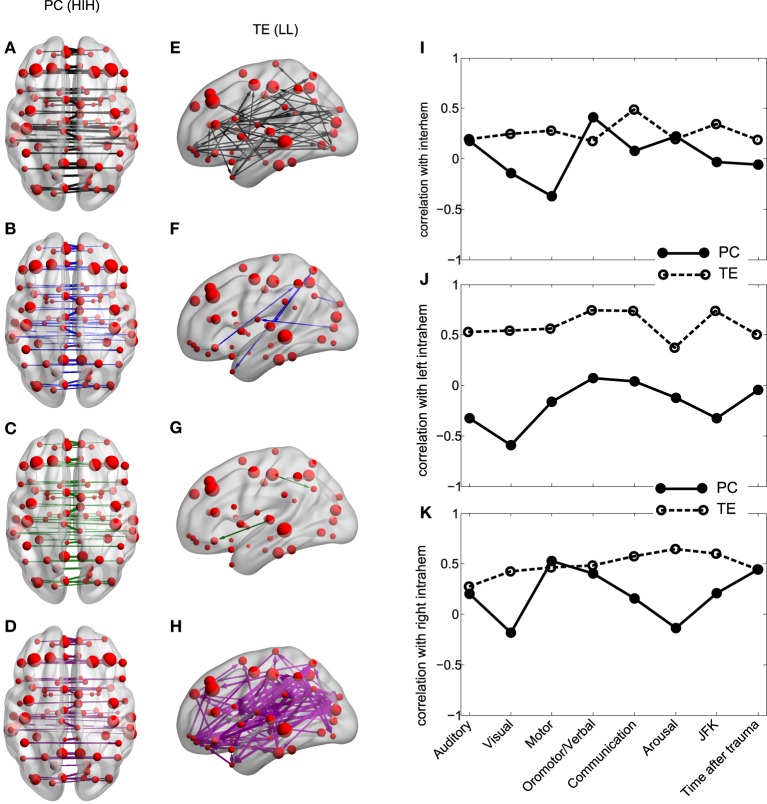
**Inter-hemispheric PC and left intra-hemispheric TE. (A–H)** PC and TE values for all the 4 different groups. The thickness of links and arrows are proportional to the PC and TE values; the thickness normalization factor is common among all the 4 groups. **(A,E)** group G1, black, **(B,F)** group G2, blue, **(C,G)** group G2a, green, **(D,H)** group G2b, magenta. **(A–D)** Visualization of the PC values HIH (homologue inter-hemispheric pairs). **(E–H)** TE in LL (left intra-hemispheric pairs). For clarity in the visualization, links have been thresholded and only TE values bigger than *TE* = 0.2 are depicted. **(I–K)** Correlation between PC (solid line) and TE (dashed) with the CRS-R scores at the different functional scales: Auditory, Visual,Motor, Oromotor/Verbal, Communication, Arousal and the total sum over all the function scales (JFK) as well as with the acquisition time after trauma. The correlation has been calculated over pairs which are **(I)** inter-hemispheric (HIH for PC and (HLR+HRL)/2 for TE, **(J)** left intra-hemispheric (LL) and **(K)** right intra-hemispheric (RR).

The results show that for all analyzed areas the best two discriminators are the between-homologue inter-hemispheric (HIH) PC (Figures 4A–D) and the left intra-hemispheric (LL) TE (Figures 4E–H). Here, colors denote group differences: black (G1), blue (G2), green (G2a) and magenta (G2b). For both PC and TE the thickness of links and arrows is proportional to the PC and TE values.

For PC there is a manifest anatomical disparity in the correlations pattern: it can be observed that homologue areas that are closer to each other show stronger correlations than farther ones (i.e., thicker connections at shorter distances in comparison with thinner connections at longer distances). To disentangle the behavior of the neural correlations regarding to a spatial factor, we look at the Euclidean distances between the centroids of homologue areas. For G1 the areas close to each other presented a high correlation, and beyond a threshold distance of 20 mm, correlations decreased, although the values remained high. Interestingly, the same behavior was found in G2. However, the correlation values there were shifted down, with lower mean value for areas closer than 20 mm, and decreasing for increasing distances. Thus, for ROIs areas distance-separated smaller than 20 mm, differences between G1 and G2 were smaller compared to areas separated at long distances, distance separation <20 mm pval = 10^−6^, distance >40 mm pval = 10^−14^. When inspecting G2a and G2b subgroups, there were no observable differences for anatomically closer areas, whilst it could be detected a higher correlation of some of the anatomically further areas for G2b.

Regarding to the TE, not only the mean values of TE in LL areas were different between groups (Table 3), but the number of significant values of TE, i.e., the number of arrows plotted in Figures 4E–H varies across different groups. This number was more than 9 times bigger in G1 compared with G2 (G1 # links = 47, Figure 4E; G2 # links = 5, Figure 4F). When comparing with group G2b, this number doubled the one in group G1 (# links = 99, Figure 4H), possibly indicating a “transient” brain state in the pattern of information flows in group G2b in comparison with control.

### 3.4. Correlation between fMRI measures and CRS-R scores

We then asked if the two fMRI measures, between-homologue inter-hemispheric PC and left intra-hemispheric TE were correlated with the neurological and behavioral scale given by the CRS-S. This is represented in Figures 4I–K. For homologue inter-hemispheric pairs we found that TE gave the biggest correlation with the corresponding value in the communication function scale. For left intra-hemispheric pairs, TE had 0.73 correlations with oromotor/verbal function scale, 0.73 with the communication function scale and 0.73 with the total CRS-R (marked as “JFK” in Figures 4I–K).

## 4. Discussion

In this study we have investigated whether the functional connectivity is altered as a consequence of consciousness disturbances. We have applied the PC and the TE approaches to analyze the FC from resting-state fMRI data. We have compared two groups, healthy subjects and Disorder of Consciousness patients. The analysis was done over the 90 anatomical brain areas, defining regions of interest from the AAL atlas. We have grouped the different pairs of ROIs in inter-hemispheric homologue regions, inter-hemispheric, left intra-hemispheric, right intra-hemispheric and total (all regions). We have found two particular markers that account for the large-scale disturbance of patients brain function: the PC calculated over homologue inter-hemispheric (HIH) regions and the TE calculated over the left intra-hemispheric (LL) ROIs.

The PC in HIH regions was found to be notably larger for control compared to DOC patients. This results holds also when comparing G1 with the recovered G2b group. The same comparison but done over the total average of the 90 regions did not shown significant differences. Thus, one relevant result of our analysis is the finding that only by the calculation of the PC in the proposed grouping of brain regions, it was possible to detect a significant marker for the patients disturbance, results that is hidden when we looked at the PC of the total AAL brain regions.

In the case of TE, the total score did not show any significant difference either, but the brain subdivision revealed that the intra-hemispheric influences were different in control respect DOC. This happened for both LL and RR, although the TE in LL discriminated better than in RR. This is a very novel finding whose origin is still unclear and deserves further investigation.

### 4.1. Methodological issues

The PC is a straightforward measure able to eliminate for each specific ROIs pair, the contribution to the correlations coming from common neighbors, preserving *effective* correlations between two time series. Unlike the PC which is a symmetrical measure, the TE quantifies interaction between ROIs in a directed form, i.e., region A influences to region B but the opposite is not necessary true. In concrete, TE quantifies information bits (uncertainty reduction) flowing from one ROI to the future of the other. For the case of Gaussian data, the information bits measured by the TE coincide with the Granger causality measured from time series (Barnett et al., 2009); however for non Gaussian data, TE and causality might result in different measures.

TE emerges as a very suitable measure for the study of temporal causality in brain fMRI activity in parallel to the advantage of an accurate spatial resolution. TE assessment in a population of patients with disorder of consciousness provides the opportunity of gaining insight into brain mechanisms of information processing and the finding of possible predictors of coma outcome.

Regarding to the calculation of TE, it is well-known that the computation of the entropies with small data sets introduces some a bias (Panzeri and Treves, 1996; Paninski, 2003; Bonachela et al., 2008). Because we are performing groups comparison with the same data size in each group (i.e., the time series in each subject have the same data points), such a bias will be the same in the two groups, thus not affecting the validity of the groups comparison. Nevertheless, as far as we understand there is not any reported study analyzing either information reduction (i.e., TE) or causality in fMRI data from DOC patients.

### 4.2. Inter-relation between PC and TE in DOC patients

To exhibit high correlations is different from having high TE between two time series. This can be clearly understood by a counter-example; two fully correlated time series have zero TE as to compute the uncertainty reduction in the future of *i*, conditioning on the two pasts *i* and *j* is not adding any further information to the situation of solely adding the past of *i*, i.e., the two terms in the right-hand side in Equation (2) are equal. As a consequence of this, the observation of having high PC for HIH pairs in healthy subjects implies to have high isolation of the information within hemispheres; thus, the TE values in both LL and RR are significantly higher than the corresponding values in HLR and HRL.

Interestingly, we found that while PC is reduced in DOC patients between inter-hemispheric homologue areas, TE shows an altered pattern at the level of general inter-hemispheric interactions. In the control group we observe that despite the coherence is high between homologue areas, their TE is low. Conversely, while PC between hemispheres is low, LR and RL TE are high. The DOC patients show the same trend, although the LR and RL TE is significantly lower than in controls. This supports the notion that consciousness arises from long-range modulation of neural activity. A disruption in long-range communication could affect mechanisms such as increase of stimulus' salience, facilitation of propagation across sparsely connected networks, and selective routing (Ganzetti and Mantini, 2013), mechanisms that are related to conscious processing (Gaillard et al., 2009).

### 4.3. rs-fMRI inter-hemispheric correlations and gamma rhythms

Recently it has been shown that the inter-hemispheric correlations in the rs-fMRI dynamics correlate with the inter hemispheric coherence exhibited by electrophysiological recordings in human sensory cortex (Nir et al., 2008), mainly with the slow modulation of the gamma rhythms in Local Field Potentials. Other studies have also found such modulation in high-level cognition tasks (Vidal et al., 2012). Thus, one could conjecture that at the functional level, a breakdown in the inter-hemispheric rs-fMRI correlations in DOC patients could be an indication of a similar deficit in the gamma power coherence. One possibility is that low-frequency oscillatory activity is related to an underlying neuronal mechanism allowing for maintenance and consolidation of neural events across wide sections of the brain, and for the handling of incoming stimuli (de Pasquale, 2010; de Pasquale et al., 2012). Although increasing evidence points toward a property of the brain relevant for conscious processing, Vidal et al. (2012) point out that gamma-amplitude correlation would also be reflecting the parallel organization of the brain, where neural networks interact for purposeful processing of information.

### 4.4. Comparison with previous results

As fas as we know, a single study have reported that DOC patients in comparison with healthy subjects manifest a strong reduction in the inter-hemispheric correlations in the rs-fMRI time series (Ovadia-Caro et al., 2012). The authors in (Ovadia-Caro et al., 2012) did not use any atlas to compute inter-hemispheric correlations; instead they investigated specific areas such as pre- and post-central gyrus and the intra-parietal sulcus. Among other reasons, the authors selected those areas for being well separated each from the other (arguing the existence of less noise in the signal). This is consistent with our finding that DOC patients kept more similar correlations to control for ROIs separation below 20 mm. In addition to this, our study adds the novelty of having analyzed the FC obtained by the TE.

### 4.5. TE density to measure consciousness alteration

We have shown in Figures 4E–H how the number of TE connections can account not only for the differences between control (G1) and DOC (G2) but for the transitory brain state in the group G2b: the patients that awaked and became fully conscious at the second fMRI acquisition. Thus, we have found that the number of TE connections were 47 (G1), 5 (G2) and 99 (G2b). In a similar spirit, Seth et al. (2006) defined the causal density for measuring consciousness in brain states as the number of Granger-causality connections flowing in and out per each specific area. Interestingly, a similar behavior has been reported during recovery from anesthesia, where an increment in functional connectivity above the normal wakeful baseline is found (Hudetz, 2012).

### 4.6. DOC impairment at specific brain areas

The aim of this analysis is not to work at the level of an individual DOC patient but to search for rs-fMRI markers that can account for groups differences in DOC patients. We have not studied yet any measure that can account for DOC impairment at specific brain areas. To this end, one could study in principle the FC graphs obtained by either PC or TE using complex networks analysis, or any other kind of graph exploration methods. In a much simpler spirit (just to illustrate that this approach is plausible), we have chosen to plot the PC values per area comparing group G2 versus G1. This is illustrated in the Figure S1. The decorrelation index per area is plotted, (corrG1-corrG2)/corrG1. Colored in blue, the five biggest decorrelation indices correspond to the following areas: Fusiform, Insula, Parietal Superior, Precentral and Temporal Superior, revealing that those areas had the major DOC impairment. Conversely the areas with less DOC impairment (colored in red) were the Cingulum Anterior, Cingulum Middle, Frontal Superior Orbital, Superior Motor Area and Temporal Inferior.

### 4.7. Limitations of the study

One of the important limitation of studying DOC patients is the great amount of involuntary movements they exhibit, leading to potential artifacts in the fMRI acquisition. Techniques to overcome this issue include affine transformations to the time series creating a head-motion parameter matrix which can be used to regress out and remove the spurious variances they introduce (Fox et al., 2005). Although these methods can correct signals from movements spanning the dimensions of up to 3–4 voxels, recent work (Power et al., 2012) suggest that no technique could remove completely the effects of these artifacts over the FC. Thus especial care is necessary to tackle these problems and, eventually, discard the entire scan.

### 4.8. Future directions

In this study PC and TE measures were used to assess for the assessment of functional connectivity in unconscious patients. In particular we characterized their disruptions at an anatomical level, in the basis of distances between homotopic areas. Other questions that can be explored, include the integrity of FC between the areas that constitute hubs in the brain network, between areas with high *rich-clubness* (van den Heuvel and Sporns, 2011), or between associative vs. sensory areas.

## Funding

Jesus M. Cortes is supported by Ikerbasque: The Basque Foundation for Science. Jesus M. Cortes acknowledges financial support from Junta de Andalucia, grant P09-FQM-4682. Verónica Mäki-Marttunen, Mirta Villarreal, and Dante R. Chialvo are partially supported by CONICET (National Council of Scientific and Technological Research) of Argentina. Additional support was provided by the Department of Neurology, and Department of Teaching and Research of FLENI, Buenos Aires, Argentina.

### Conflict of interest statement

The authors declare that the research was conducted in the absence of any commercial or financial relationships that could be construed as a potential conflict of interest.

## Supplementary material

Figure S1 **DOC impairment evaluated at specific brain areas. (A)** Decorrelation indices defined as (corrG1-corrG2)/corrG1 computed for each of the different 45 brain areas. In blue, top-five values of decorrelation index; in red, bottom five (positive) values. **(B)** Scatter of between-homologue inter-hemispheric correlations G1 vs. G2, each point represents one of the 45 brain areas.

## Abbreviations

fMRI: functional Magnetic Resonance Imaging
rs: resting state
DOC: disorder of consciousness
BOLD: Blood-Oxygen-Level-Dependent
FC: Functional Connectivity
TE: Transfer Entropy
PC: Partial Correlation.
