# Supplementary material for: Disruption of transfer entropy and inter-hemispheric brain functional connectivity in patients with disorder of consciousness
Source: Front Neuroinform. 2013 Nov 13;7:24. doi: 10.3389/fninf.2013.00024 (PMC3826091; doi:10.3389/fninf.2013.00024)
Supplement: Supplementary file 2 [file DataSheet1.PDF]

Table S1. PC average values ± standard deviation thresholded at 5% confidence. **significantly different from G1; p<0.05.* Significant differences are indicated with black asterisks for ANOVA and green for Kruskal-Wallis tests. LR: inter-hemispheric; HIH: between-homologue inter-hemispheric ; LL: left intra-hemispheric; RR: right intra-hemispheric.

PC	G1	G2	G2a	G2b
LR	0.10±0.004	0.11±0.008	0.10±0.009	0.11±0.002 *
HIH	0.41±0.031	0.24±0.041 * *	0.24±0.045 * *	0.25±0.053 * *
LL	0.11±0.006	0.12±0.008 *	0.12±0.008 *	0.12±0.009 *
RR	0.11±0.007	0.12±0.010 *	0.12±0.011 *	0.12±0.003 *
Total	0.11±0.004	0.11±0.008 * *	0.11±0.008 *	0.12±0.003 * *
