# Supplementary material for: Disruption of transfer entropy and inter-hemispheric brain functional connectivity in patients with disorder of consciousness
Source: Front Neuroinform. 2013 Nov 13;7:24. doi: 10.3389/fninf.2013.00024 (PMC3826091; doi:10.3389/fninf.2013.00024)
Supplement: Supplementary file 3 [file DataSheet2.PDF]

Table S2. PC average values ± standard deviation thresholded at 100% confidence (i.e., zero threshold); **significantly different from G1; p<0.05.* Significant differences are indicated with black asterisks for ANOVA and green for Kruskal-Wallis tests. LR: inter-hemispheric; HIH: between-homologue inter-hemispheric ; LL: left intra-hemispheric; RR: right intra-hemispheric.

PC	G1	G2	G2a	G2b
LR	0.16±0.003	0.16±0.006	0.16±0.007	0.17±0.002 * *
HIH	0.42±0.028	0.28±0.036 * *	0.28±0.034 * *	0.29±0.044 * *
LL	0.16±0.005	0.17±0.007 * *	0.17±0.006 * *	0.17±0.008 * *
RR	0.16±0.005	0.17±0.008 * *	0.17±0.008 * *	0.17±0.004 * *
Total	0.16±0.003	0.17±0.006 * *	0.17±0.006 * *	0.17±0.003 * *
