# Supplementary material for: Disruption of transfer entropy and inter-hemispheric brain functional connectivity in patients with disorder of consciousness
Source: Front Neuroinform. 2013 Nov 13;7:24. doi: 10.3389/fninf.2013.00024 (PMC3826091; doi:10.3389/fninf.2013.00024)
Supplement: Supplementary file 4 [file DataSheet3.PDF]

Table S3. TE average values ± standard deviation thresholded at 5% confidence. **significantly different from G1; p<0.05*. Significant differences are indicated with black asterisks for ANOVA and green for Kruskal-Wallis tests. HLR: homologous inter-hemispheric from left to right; HRL: homologous inter-hemispheric from right to left; LL: left intra-hemispheric; RR: right intra-hemispheric; LR: inter-hemispheric left to right; RL: inter-hemispheric right to left.

TE	G1	G2	G2a	G2b
HLR	0.003±0.014	N/A	N/A	N/A
HRL	0.006±0.017	0.008±0.024	0.004±0.013	N/A
LL	0.020±0.015	0.007±0.007 * *	0.004±0.004 * *	0.016±0.003
RR	0.019±0.013	0.013±0.021 *	0.004±0.004 * *	0.032±0.031
LR	0.020±0.015	0.008±0.010 * *	0.004±0.004 * *	0.019±0.011
RL	0.020±0.014	0.014±0.025 *	0.005±0.006 * *	0.026±0.021
Total	0.020±0.012	0.010±0.013 * *	0.004±0.003 * *	0.023±0.015
