# Supplementary material for: Disruption of transfer entropy and inter-hemispheric brain functional connectivity in patients with disorder of consciousness
Source: Front Neuroinform. 2013 Nov 13;7:24. doi: 10.3389/fninf.2013.00024 (PMC3826091; doi:10.3389/fninf.2013.00024)
Supplement: Supplementary file 5 [file DataSheet4.PDF]

Table S4. TE average values $\pm$ standard deviation thresholded at 100% confidence (i.e., zero threshold); **significantly different from G1; p<0.05*. Significant differences are indicated with black asterisks for ANOVA and green for Kruskal-Wallis tests. HLR: homologous inter-hemispheric from left to right; HRL: homologous inter-hemispheric from right to left; LL: left intra-hemispheric; RR: right intra-hemispheric; LR: inter-hemispheric left to right; RL: inter-hemispheric right to left.

TE	G1	G2	G2a	G2b
HLR	1.808 ±0.154	1.392±0.397 * *	1.266 ±0.400 * *	1.729±0.302
HRL	1.793±0.163	1.444±0.475 * *	1.288±0.463 * *	1.783± 0.378
LL	1.933±0.182	1.439±0.439 * *	1.297±0.430 * *	1.844±0.374
RR	1.923±0.187	1.471±0.492 * *	1.311±0.483 * *	1.859±0.400
LR	1.938±0.184	1.433±0.431 * *	1.295±0.427 * *	1.832±0.362
RL	1.924±0.190	1.486±0.506 * *	1.318±0.490 * *	1.886±0.425
Total	1.929±0.183	1.457±0.464 * *	1.305±0.454 * *	1.855±0.389
