# Supplementary material for: Disruption of transfer entropy and inter-hemispheric brain functional connectivity in patients with disorder of consciousness
Source: Front Neuroinform. 2013 Nov 13;7:24. doi: 10.3389/fninf.2013.00024 (PMC3826091; doi:10.3389/fninf.2013.00024)
Supplement: Figure S1 — DOC impairment evaluated at specific brain areas. (A) Decorrelation indices defined as (corrG1-corrG2)/corrG1 computed for each of the different 45 brain areas. In blue, top-five values of decorrelation index; in red, bottom five (positive) values. (B) Scatter of between-homologue inter-hemispheric correlations G1 vs. G2, each point represents one of the 45 brain areas. [file Presentation1.PDF]

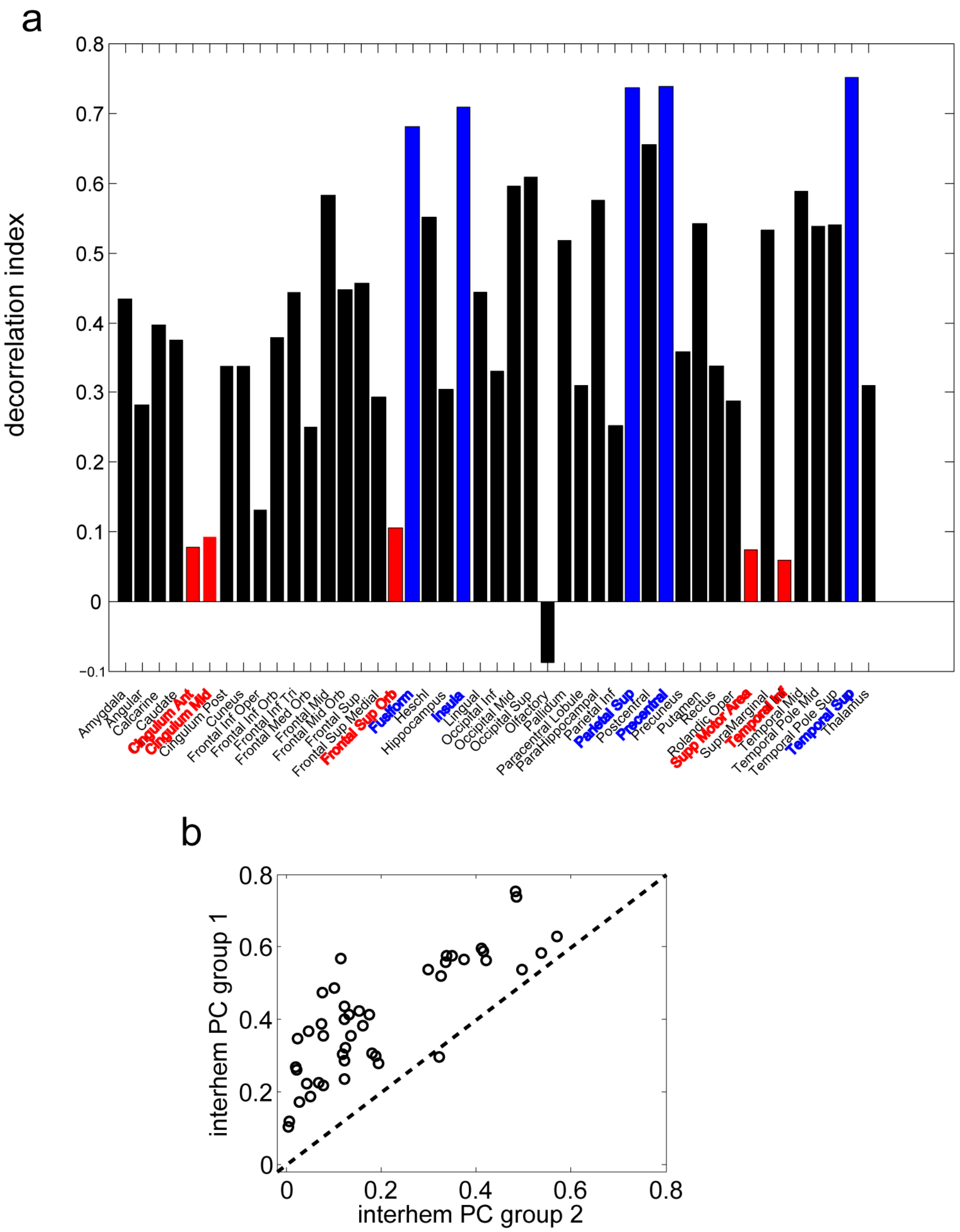

Figure S1. DOC impairment evaluated at specific brain areas a: Decorrelation indices defined as (corrG1-corrG2)/corrG1 computed for each of the different 45 brain areas. In blue, top-five values of decorrelation index; in red, bottom five (positive) values. **b**: Scatter of between-homologue inter-hemispheric correlations G1 vs G2, each point represents one of the 45 brain areas.
